# Morphometrics in three dimensional choroidal vessel models constructed from swept-source optical coherence tomography images

**DOI:** 10.1038/s41598-022-17039-9

**Published:** 2022-09-06

**Authors:** Yukinori Sugano, Shunsuke Maeda, Yutaka Kato, Akihito Kasai, Shingo Tsuji, Masahiro Okamoto, Tetsuju Sekiryu

**Affiliations:** 1 Department of Ophthalmology, Fukushima Medical University, Fukushima, Japan; 2 Department of Cellular and Integrative Physiology, Fukushima Medical University, Fukushima, Japan; 3 Department of Systems Neuroscience, Fukushima Medical University, Fukushima, Japan

**Keywords:** Medical research, Biomarkers, Diagnostic markers

## Abstract

We created three types of vessel models: vessel volume, surface, and line models from swept-source optical coherence tomography images and tested experimentally calculated three-dimensional (3D) biomarkers. The choroidal volume (CVolume), surface area (VSurface), and vessel length-associated index (VLI) were measured. The calculated 3D parameters were the mean choroidal thickness, choroidal vascularity index (CVI), vessel length density index (VLDI), vessel length to the stromal (VL–S) ratio, surface-to-volume ratio (S–V ratio), and vessel diameter index (VDI). Cluster analysis showed that the parameters were classified into two clusters: one was represented by the VVolume including the CVolume, VSurface, CVI, S–V ratio, VLI, VDI, and subfoveal choroidal thickness and the other by the VL–S ratio including the VLDI. Regarding the regional distribution, the VVolume, CVolume, VSurface, CVI, VLI, VL–S ratio, and VDI at the foveal center were higher than at the parafovea (P < 0.01). Although the VVolume decreased with age and axial length (AL) elongation, the association of the 3D parameters with age and AL elongation differed. The VLI, VLDI, VL–S ratio, and CVI decreased with age (P < 0.01) but not with AL elongation. The results suggested a structural difference in the choroidal vessel volume reduction between aging and AL elongation. The 3D parameters may provide additional information about the choroidal vasculature.

## Introduction

The choroid is a vascular tissue between the retina and sclera, the main function of which is to supply oxygen and nutrients to the photoreceptor cells^1,2^. Previous reports have suggested that disorders of the choroidal vasculature may be associated with macular diseases^3–7^. The choroidal vasculature has been evaluated in vivo using indocyanine green angiography (ICGA). While ICGA visualizes choroidal circulatory disorders, the structural evaluation is limited because of its 2-dimensional (2D) nature and potential dye toxicity. Optical coherence tomography (OCT), a non-invasive imaging technology, visualizes the choroidal structure^8^. The choroidal vasculature can be evaluated by measuring the area of dark silhouettes in the OCT choroidal images^9^. In the early stage of choroidal image analysis using OCT, the success rate of the volume scan was low in visualizing three-dimensional (3D) structures because of the slow scanning speed of the OCT machines^10^. Swept-source OCT (SS-OCT), which has high-speed scanning and a long-wavelength light source, provides the volume data in the choroidal images in a wide area. 3D visualization of the choroid has become a clinically available OCT technique to facilitate choroidal vessel assessment.

Improved choroidal imaging parameters have contributed to understanding the choroidal pathophysiology^4,6^ and clinical evaluation of diseases^5,11,12^. The choroidal thickness and choroidal vessel ratio to the choroidal volume, i.e., the choroidal vascularity index (CVI), have been well investigated^13–17^. The previous reports have suggested that aging and long axial length (AL) were associated with a thin choroid and low CVI^9,13^. Clinically, the choroidal thickness can be used as a clinical indicator for treating Vogt–Koyanagi–Harada disease^18^. The CVI changes with anti-vascular endothelial growth factor treatment for age-related macular degeneration^19^.

The choroidal volume and CVI also were evaluated in 3D choroidal images and the relation to age and AL was suggested^20,21^. We recently reported a reconstructed 3D model of the choroidal vasculature using SS-OCT data^22,23^. A modelized vessel allows morphologic measurements. Although the 3D vessel models potentially provide new information about the choroidal vasculature, the feasibility and validity of the 3D choroidal vessel model measurements are not established.

In this study, we created three types of 3D choroidal vessel models: vessel volume, surface, and line models, assuming the path of the vessels from the SS-OCT volume images. We also measured each model and experimentally calculated new biomarkers. The correlations of each parameter, regional distribution, and the relation to age and AL were examined.

## Methods

The Institutional Ethics Committee of the Fukushima Medical University approved this prospective, noncomparative case series, which was conducted according to the tenets of the Declaration of Helsinki.

### Participants

From February to May 2020, 77 individuals older than 18 years with no history of ophthalmologic diseases or systemic diseases except for controlled hypertension were recruited by advertising and included in this study after providing written informed consent. We examined one eye of each participant based on their preference. Cases with an AL of 26.5 mm or more and 21 mm or less were excluded. Participants were required to undergo a normal ocular examination and spectral-domain OCT screening of the macula (Spectralis-™, Heidelberg Engineering, Heidelberg, Germany).

### Image acquisition

The images were acquired using a SS-OCT device (PLEX Elite 9000; Carl Zeiss Meditec, Dublin, CA) without mydriatics. The exported raw data from the structural OCT images (500 × 1536 × 500 pixels, width × height × depth) were converted to 500 × 256 × 500 pixel images corresponding to 6 × 3 × 6 mm in isometric physical volume using Fiji software^24^. The AL was measured during the same session using the IOLMaster 700 (Carl Zeiss Meditec, Dublin, CA).

### Image processing

Choroidal volume and choroidal vessel segmentation were performed in two steps. First, the choroidal volume was segmented using a convolutional neural network (CNN) (MATLAB R2020a)^23^. Second. The vessels were segmented by applying three-dimensional multiscale Hessian enhancement for an OCT stack image after image intensity normalization^22^. The 3D choroidal vessels were made by masking the vessel image outside the choroidal volume made in the previous step. These source codes were opened on Github (https://github.com/FmuOphthalOctChoroidBloodVessels/vesselnessfilter, https://github.com/FmuOphthalOctChoroidBloodVessels/chroidsegmentation).

The details of the image processing steps are described below.

#### Choroidal volume segmentation

The methods of choroidal segmentation were previously reported^23^. In short, we trained the VGG-19 CNN (MATLAB R2020a) using 7000 choroidal slices to detect the line of the RPE and the C/S border. One retina specialist manually labeled the choroidal boundary on the SS-OCT images. The choroidal boundary was defined as the distance between the area beneath the retinal pigment epithelium (RPE) to the choroid/scleral border (C/S border), including the posterior stroma. The choroidal border was extracted using the CNN in each slice. We inspected all files of the automatically segmented volume scan. If we found incorrect segmentation lines, one grader who made segmentation labels for training data manually drew the correct lines. Based on a previous report, a cubic smoothing spline was applied to determine the C/S border. We calculated the choroidal volume in 500 slices.

#### Choroidal vessel segmentation

The structural OCT images were inverted between black and white. Three-dimensional multiscale Hessian enhancement was applied to the volume scan as reported previously^22^. The volume data were converted to 8 bits grayscale. The choroidal vessel volume in the choroidal volume was calculated after binarization at the fixed value after masking the choroidal volume.

#### Line model

**Figure 1:**
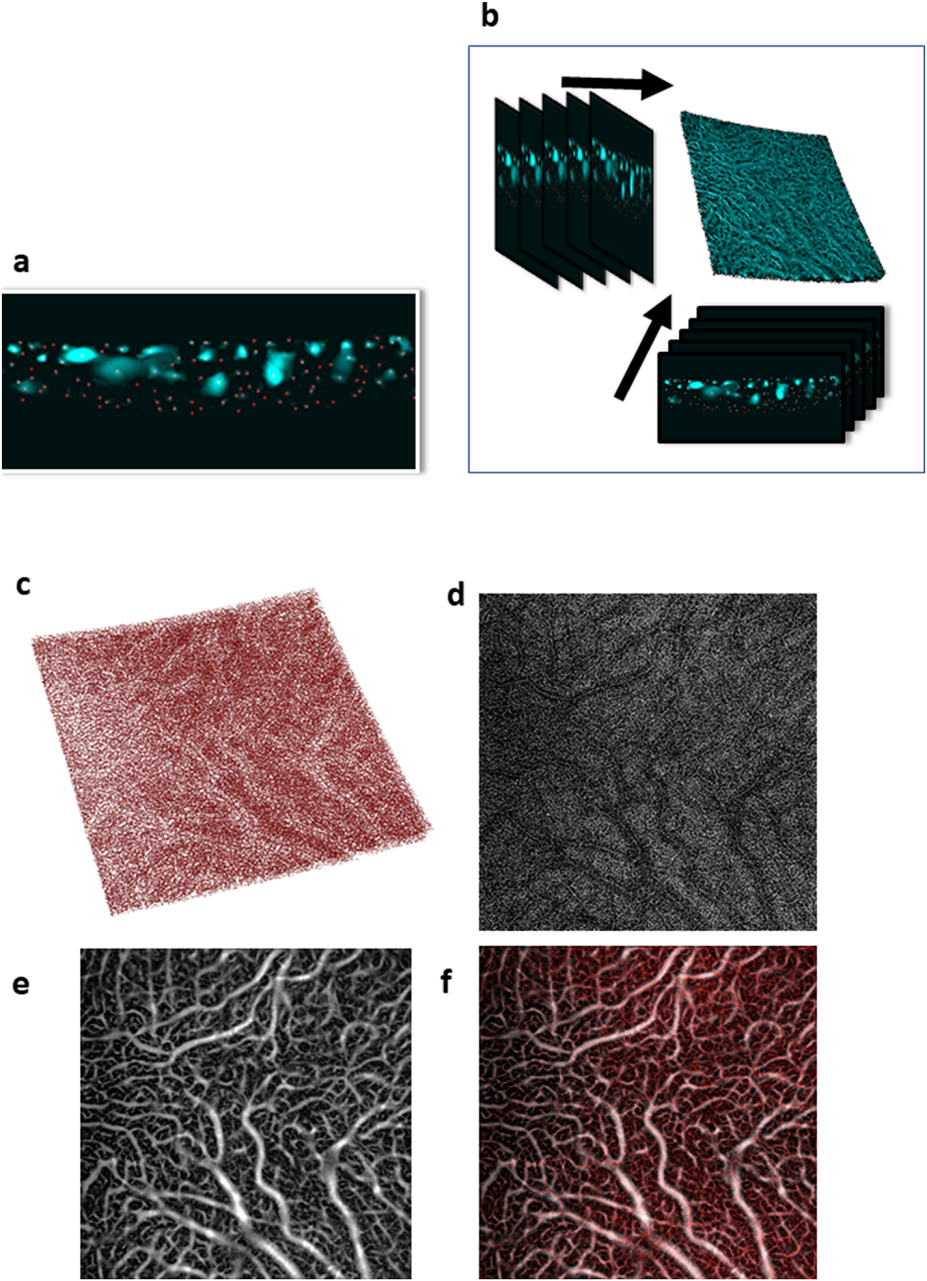
Vessel line visualization. The local high-intensity points are detected using the ImageJ function, “Find Maxima” for each slice after multiscale Hessian enhancement (**a**). The same procedure is repeated along the x-axis and z-axis. Both images are combined to make a three-dimensional line model (**b**). A three-dimensional view of the centreline reconstruction model (**c**). An en-face projection image of the centreline reconstruction model (**d**). An en-face projection image of the choroidal vasculature (**e**). A composite image of B and C (**f**).

We were able to find a peak value in the sectioned vessels in an OCT slice after the processing by the multiscale Hessian method. We detected the local peak intensity corresponding to the vessel cross-section's peak value (“Find Maxima” in Fiji)^24^. The process was repeated in each slice along the x- and z-axes (Fig. 1a,b). To make a line model, we combined two volume images along the x- and z-axes. Examples of the vessel volume and line model are shown in Fig. 1c–f. This experimentally constructed line model may provide information on vessel length independent of vessel diameter.

#### Surface model

To make a surface model, first, the edge around the voxel in all six directions was detected in the volume scan after binarization following multiscale Hessian enhancement. The number of the edge voxels in the volume was then calculated as the surface area. The border of the volume scan was not counted as the surface area.

### Region of interest (ROI) setting

**Figure 2:**
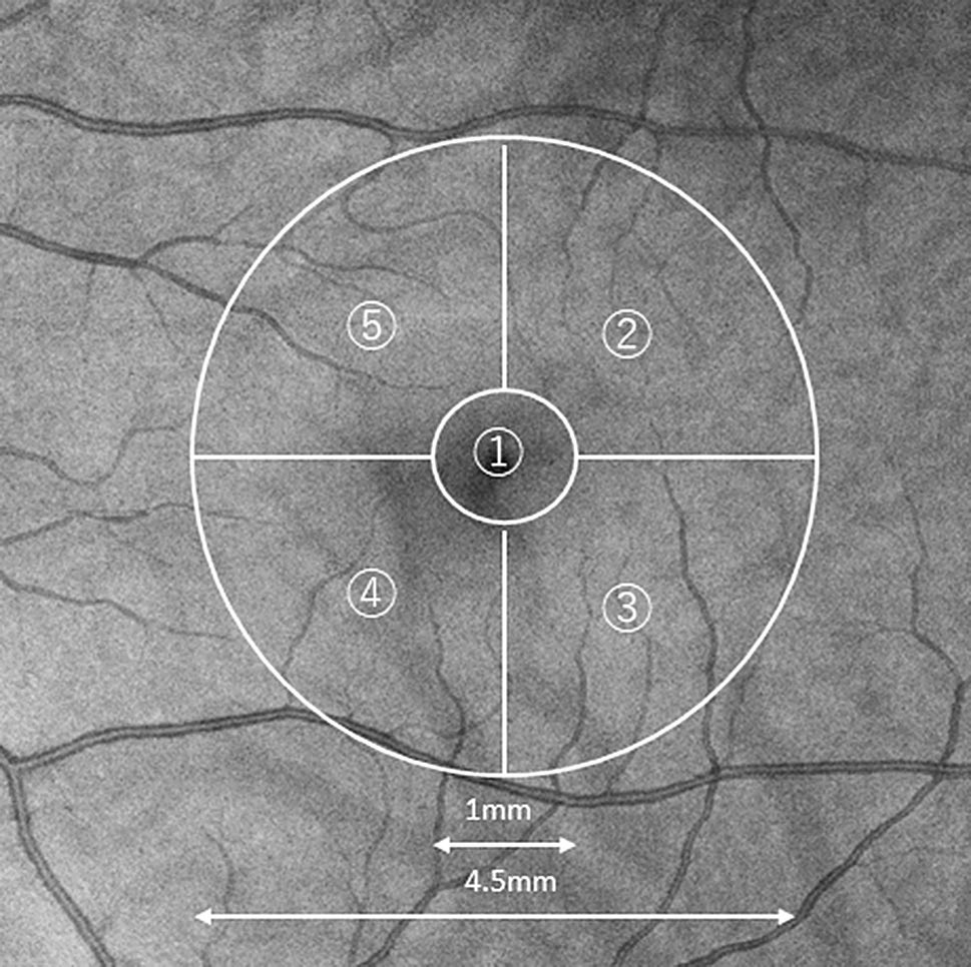
The analysis area is within a 4.5-mm circle centred on the fovea. The area is divided into five regions; ①, a circle with a 1-mm centre of the fovea and ②–⑤, outside the 4.5-mm circle except that the centre circle is divided by horizontal and vertical lines through the fovea. ②, upper temporal quadrant. ③, Lower temporal quadrant. ④ Lower nasal quadrant. ⑤ Upper nasal quadrant.

The foveal center was identified manually on the en-face projection images of the structural OCT. In practice, we detected the foveal center using an orthogonal view of the 3D volume images. First, we positioned the cursor around the foveal center. We then determined the thinnest part of the fovea along with the x-axis slice. We then moved the cursor at the thinnest part of the fovea along with the y-axis slice. Finally, we found the thinnest point of the fovea in the en-face image and read the x and y positions on the en-face image. The mean value along the x- and y-axis of the intraclass correlation coefficient determined by two graders was 0.978 in the preliminary experiment. We evaluated five zones in the ROI using a grid centered on the fovea, i.e., a central 1-mm circle and four sectors in a 4.5-mm circle outside the central 1-mm circle (parafovea) (Fig. 2).

### Parameters

Each parameter was calculated in the assigned area of the ROI. The subfoveal choroidal thickness (SFCT) and the following were calculated from the measured values of each model:

1. SFCT (mm): the length between Bruch's membrane to the C/S border.
2. Choroidal volume (CVolume) (mm^3^): the voxel numbers × 12 × 10^−3^ (mm) × 12 × 10^−3^ (mm) × 11.72 × 10^−3^ (mm).
3. Mean choroidal thickness (MCT) (mm): the CVolume/ROI area (mm^2^).
4. Choroidal vessel volume (VVolume) (mm^3^): the voxel numbers in choroidal vessel images × 12 × 10^−3^ (mm) × 12 × 10^−3^ (mm) × 11.72 × 10^−3^ (mm).
5. Vessel surface (VSurface) (mm^2^): Voxel numbers in the vessel surface model × [12 × 10^−3^ (mm) × 12 × 10^−3^ (mm) × 11.72 × 10^−3^ (mm)]^2/3^.
6. Vessel line index (VLI) (mm): the voxel numbers in the vessel line model × [12 × 10^−3^ (mm) × 12 × 10^−3^ (mm) × 11.72 × 10^−3^ (mm)]^1/3^.
7. Choroidal vascularity index (CVI): VVolume/CVolume.
8. Vessel length density index (VLDI) (mm^−2^): VLI/VVolume.
9. Vessel length to stroma volume ratio (VL–S ratio) (mm^−2^): VLI/(CVolume − VVolume).
10. S–V ratio (mm^−1^): VSurface/VVolume.
11. Vessel diameter index VDI (mm): (VVolume/VLI)^1/2^.

### Statistical analysis

Statistical analyses were performed using the JMP Pro 15 (SAS Institute, Cary, NC). The Wilcoxon rank-sum test compared the MCT, CVI, VLDI, VL–S ratio, S–V ratio, and DI between each ROI. The correlation was calculated by Spearman's correlation analysis. P < 0.05 was considered significant.

## Results

Seventy-seven eyes were examined. The demographics of the subjects are shown in Table 1. The mean patient age was 54 ± 21 years (± standard deviation). The mean spherical equivalent was − 1.72 ± 2.51 dioptres (D), and the mean AL was 24.36 ± 1.20 mm.

**Table 1:** Demographic data of the study participants.

	Mean/incidence	SD	95% CI	
Age	54	21.0	49	58
Female	41 (53%)			
Left eye	38 (51%)			
LogMAR	− 0.007	0.1	− 0.05	− 0.08
AL (mm)	24.36	1.2	24.09	24.63
Spherical equivalent (diopters)	− 1.72	2.5	− 2.29	− 1.15
Ocular pressure (mmHg)	14.8	2.0	14.4	15.3

*LogMAR* logarithm of the minimum angle of resolution, *SD* standard deviation, *CI* confidential interval.

The choroidal volume segmentation in four eyes (5.2%) resulted from a partly inadequate C/S border segmentation was corrected manually by Fiji software. The CVolume, VVolume, VSurface, and VLI within the 4.5-mm circle were, respectively, 5.469 ± 1.68 mm^3^, 2.242 ± 0.95 mm^3^, 158.2 ± 51.82 mm^2^, and 820.1 ± 178.1 mm. The MCT, CVI, VLDI, VL–S ratio, S–V ratio, and VDI were, respectively, 0.344 ± 0.11 mm, 0.396 ± 0.07, 165.3 ± 28.1 (mm^−2^), 275.6 ± 53.2 (mm^−2^), 76.32 ± 12.6 (mm^−1^), and 49.37 ± 5.58 µm^2^ within the 4.5-mm circle at the fovea. The MCT (P < 0.001), CVI (P < 0.001), VL–S ratio (P < 0.001) and VDI (P = 0.004) were high in the foveal center, but the VLDI, and S–V ratio was not (Table 2). The CVI and VDI in the upper nasal quadrant were the highest among the four quadrants (Supplement Table).

**Table 2:** Regional distribution of each measurement: the centre and the parafovea. *SD* standard deviation. P value: centre vs. parafovea.

Parameter	Mean	SD	P value
**Choroidal volume (mm**^**3**^**)**			
Center	0.286	0.09	< 0.001
Parafovea	5.183	1.59	
Total	5.469	1.68	
**MCT (mm)**			
Center	0.364	0.11	< 0.001
Parafovea	0.343	0.11	
Total	0.344	0.11	
**VVolume-I (mm**^**3**^**)**			
Center	0.122	0.05	< 0.001
Parafovea	2.120	0.90	
Total	2.242	0.95	
**VSurface-I (mm**^**2**^**)**			
Center	8.423	2.74	< 0.001
Parafovea	149.8	49.2	
Total	158.2	51.8	
**VLI (mm)**			
Center	42.9	12.3	< 0.001
Parafovea	777.2	169	
Total	820.1	178	
**VLDI (mm**^**−1**^**)**			
Center	165.9	30.4	0.942
Parafovea	165.3	28.2	
Total	165.3	28.1	
**VL–S ratio (mm**^**−1**^**)**			
Center	284.8	59.5	< 0.001
Parafovea	275.1	53.0	
Total	275.6	53.2	
**VDI (µm)**			
Center	50.38	6.02	0.004
Parafovea	49.31	5.50	
Total	49.37	5.58	
**S–V ratio (mm**^**−1**^**)**			
Center	75.43	13.0	0.251
Parafovea	76.42	12.8	
Total	76.32	12.6	
**CVI**			
Center	0.414	0.07	< 0.001
Parafovea	0.395	0.07	
Total	0.396	0.07	

**Figure 3:**
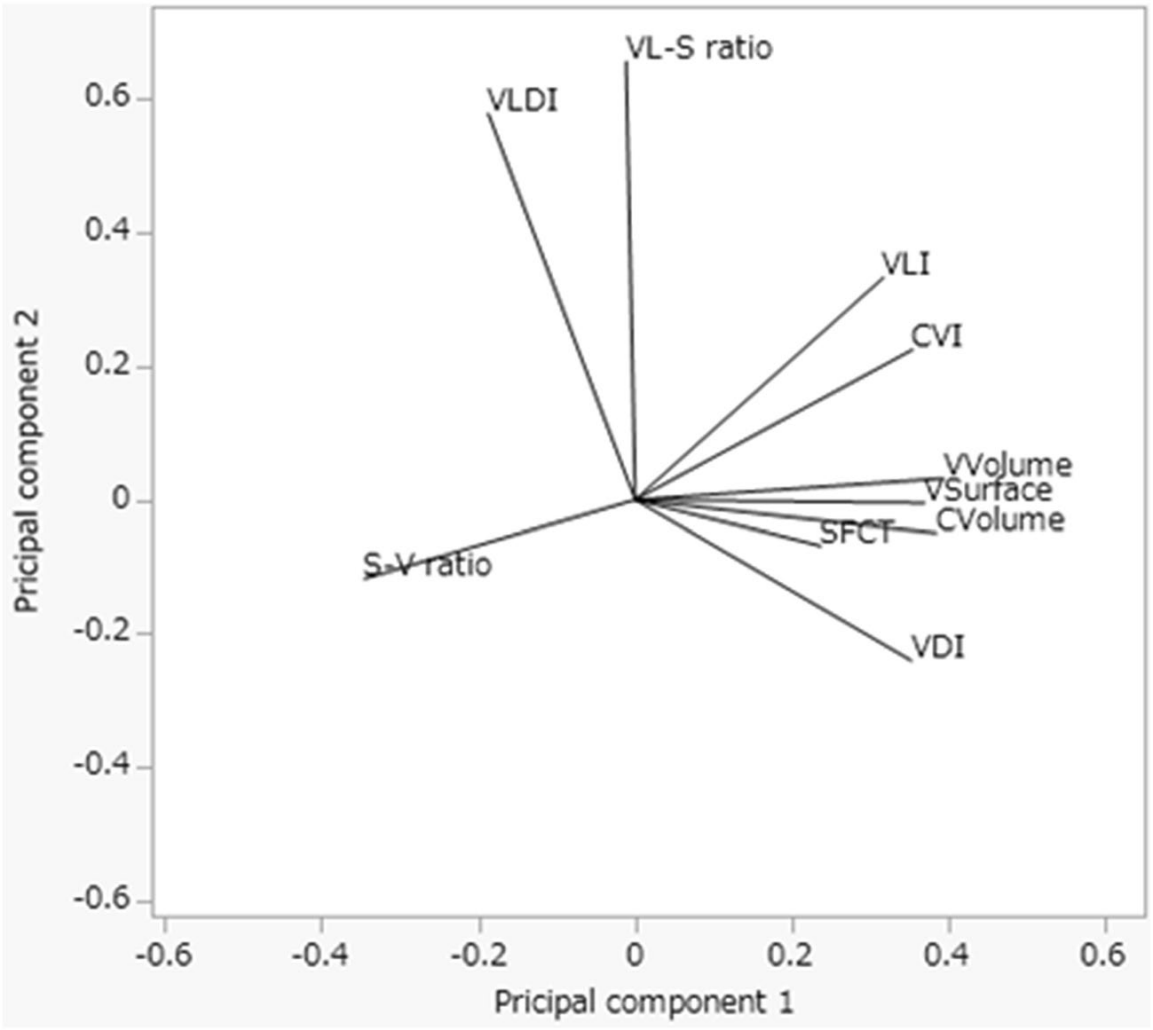
The relationship between each parameter visualized by the K means method (K = 2). Each line demonstrates the vector of each parameter.

The correlations among the parameters within the 4.5 mm circle are shown in Table 3. Seven parameters, CVolume, VVolume, VSurface, VDI, VLI, CVI, and SFCT were positively correlated with each other. The S–V ratio was correlated negatively with these seven parameters. The VLDI and VL–S ratio were correlated positively with each other. These parameters were classified into two clusters based on cluster analysis. One cluster comprised the VVolume, CVolume, VSurface, CVI, S–V ratio, VLI, VDI, and SFCT. The other included the VL–S ratio and VLDI (Table 4). The relationship is demonstrated by the biplot graph calculated by K-means cluster analysis (K = 2) (Fig. 3).

**Table 3:** The correlation table of each parameter. Bold characters indicate the correlation is P > 0.05.

	CVolume	VVolume	VSurface	VLine	VLDI	VL–S ratio	VDI	S–V ratio	CVI	SFCT
CVolume	1	–	–	–	–	–	–	–	–	–
P										
VVolume	0.975	1	–	–	–	–	–	–	–	–
P	< 0.0001									
VSurface	0.979	0.942	1	–	–	–	–	–	–	–
P	< 0.0001	< 0.0001								
VLine	0.822	0.837	0.847	1	–	–	–	–	–	–
P	< 0.0001	< 0.0001	< 0.0001							
VLDI	− 0.550	− 0.528	− 0.462	− **0.066**	1	–	–	–	–	–
P	< 0.0001	< 0.0001	< 0.0001	0.571						
VL–S ratio	**0.005**	**0.089**	**0.055**	0.464	0.717	1	–	–	–	–
P	0.966	0.442	0.638	< 0.0001	< 0.0001					
VDI	0.843	0.865	0.762	0.481	− 0.826	− 0.269	1	–	–	–
P	< 0.0001	< 0.0001	< 0.0001	< 0.0001	< 0.0001	0.018				
S–V ratio	− 0.753	− 0.856	− 0.656	− 0.655	0.488	− **0.138**	− 0.822	1	–	–
P	< 0.0001	< 0.0001	< 0.0001	< 0.0001	< 0.0001	0.231	< 0.0001			
CVI	0.778	0.888	0.727	0.788	− 0.352	0.301	0.744	− 0.947	1	–
P	< 0.0001	< 0.0001	< 0.0001	< 0.0001	0.002	0.008	< 0.0001	< 0.0001		
SFCT	0.547	0.535	0.517	0.449	− 0.355	**0.029**	0.537	− 0.491	0.483	1
P	< 0.0001	< 0.0001	< 0.0001	< 0.0001	0.002	0.801	< 0.0001	< 0.0001	< 0.0001

**Table 4:** Cluster analysis of the parameters.

Cluster	Total proportion of variation explained	Members	R square with own cluster
1	0.608	VVolume	0.975
1		CVolume	0.911
1		VSurface	0.850
1		CVI	0.823
1		S–V ratio	0.764
1		VLine	0.692
1		VDI	0.723
1		SFCT	0.344
2	0.188	VLDI	0.939
2		VL–S ratio	0.939

In univariate analysis, the CVolume (P = 0.0002), VVolume (P < 0.0001), VSurface (P = 0.0061), CVI (P < 0.0001), VDI (P < 0.0001), S–V ratio (P < 0.0001), and SFCT (P < 0.0001) decreased with age. The VLDI (P = 0.0004) and S–V ratio (P < 0.0001) increased with age. The VL–S ratio did not show statistically significant changes. The AL of the eyes within the normal range was not correlated with any parameters associated with choroidal vascular morphology (Table 5).

**Table 5:** Univariate analysis. Each parameter and age, axial length, and measurement. *SE* standard error.

Y axis	Age				Axial length			
	Estimate	SE	R^2^	P	Estimate	SE	R^2^	P
CVolume	− 0.025	0.007	0.166	0.0002	− 0.075	0.125	0.005	0.5523
VVolume	− 0.016	0.004	0.218	< 0.0001	− 0.014	0.07	0.001	0.8392
VSurface	− 0.560	0.199	0.096	0.0061	− 3.319	3.603	0.011	0.3598
VLI	− 1.700	0.968	0.039	0.0829	− 11.88	17.07	0.006	0.4883
VLDI	0.533	0.143	0.155	0.0004	− 1.715	2.703	0.005	0.5277
VL–S ratio	0.377	16.82	0.022	0.2004	− 1.299	5.115	0.001	0.8003
VDI	− 0.015	0.002	0.354	< 0.0001	0.024	0.051	0.003	0.6350
S–V ratio	0.359	0.056	0.351	< 0.0001	− 1.151	1.207	0.012	0.3430
CVI	− 0.001	0.000	0.266	< 0.0001	0.004	0.005	0.008	0.4498
SFCT	− 1.770	0.41	0.199	< 0.0001	− 0.151	7.958	0.000	0.9849

We performed multivariate analysis for each parameter. Here, the objective variable was each parameter, and the dependent variables were age and AL. All parameters, CVolume, VVolume, VSurface-I, VLI, VLDI, CVI, S–V ratio, and VDI, were significantly correlated to age. While CVolume, VVolume, VSurface-I, S–V ratio, and VDI were correlated to the AL, but the VLI (P = 0.0860), VLDI (P = 0.228), VL–S ratio (P = 0.690), and CVI (P = 0.072) were not correlated to the AL. (Table 6).

**Table 6:** Multivariate analysis. Each parameter and age, axial length.

Y axis	Age				Axial length			
	Estimate	F value	Standard β	P	Estimate	F value	Standard β	P
CVolume	− 0.035	24.73	− 0.567	< 0.0001	− 0.366	8.75	− 0.337	0.004
VVolume	− 0.022	30.82	− 0.615	< 0.0001	− 0.192	8.07	− 0.315	0.006
VSurface	− 0.839	15.02	− 0.464	0.000	− 10.207	7.39	− 0.325	0.008
VLI	− 2.610	5.81	− 0.305	0.019	− 33.323	3.15	− 0.225	0.080
VLDI	0.626	14.85	0.463	0.001	3.428	1.48	0.146	0.228
VL–S ratio	0.440	1.745	0.172	0.191	2.315	0.161	0.052	0.690
VDI	0.000	53.25	− 0.733	< 0.0001	0.000	8.46	− 0.292	0.005
S–V ratio	0.422	45.51	0.696	< 0.0001	2.317	4.55	0.220	0.036
CVI	− 0.002	30.61	− 0.612	< 0.0001	− 0.009	3.34	− 0.202	0.072
SFCT	− 2.291	25.74	− 0.577	< 0.0001	− 18.970	5.86	− 0.275	0.018

## Discussion

This study evaluated the experimental parameters derived from the 3D vessel models. The parameters were classified into two clusters represented by the VVolume and VLDI. The VVolume and associated parameters showed regional variations, but the VLDI did not. The VLDI increased with age, but not with AL elongation.

In the fields of OCT imaging, SFCT and CVI are widely used in the research of the choroid. Agrawal et al. reported that the SFCT was strongly correlated to the CVI in 2D analysis^13^, while Cheong et al. did not find a relation between the two parameters in 2D and 3D analysis^25^. The SFCT did not show a high correlation with the CVolume in this study. The dissociation between the two parameters may be caused by the regional variation of the choroidal thickness and asymmetrical shape of the choroid. The low sensitivity of CVI may also contribute to the inconsistency. The current study demonstrated the close relationship between the CVolume and the VVolume. CVI treats these two factors as a numerator and a denominator. As a result, the sensitivity to detect the CVI change may be reduced.

On the other hand, the luminal/stromal ratio of the choroid is more sensitive to detecting the difference between the two structures. The luminal/stromal ratio is strongly influenced by age and the axis of the eye^9^. Consequently, we have to examine a large number of cases classified by age and AL when comparing a disease or estimating the drug efficacy. It is worth exploring new parameters for the evaluation of the choroid.

### The relations in 3D parameters

3D choroidal vessel models can provide new parameters related to vessel morphology. We experimentally prepared new parameters from three choroidal vessel model types: S–V ratio, VLDI, VL–S ratio, and VDI. S–V ratio is one of the parameters to evaluate the structure. Smaller size objects show a greater ratio of surface area to volume. The line model was the assemblies of a single point in each section of the vessel. We assumed that the results of counting the voxels in this model might reflect the length of vessels. Then the VLDI, VL–S ratio, and VDI were assumed to reflect the total length of vessel per volume unit, the total length of vessel per volume unit of the stroma, and the diameter of the vessel, respectively.

Cluster analysis revealed that 3D model parameters were classified into two clusters represented by the VVolume and VLDI. We referred to them as the volume-associated and length-associated clusters, respectively. The VVolume, CVolume, and VSurface were highly correlated with each other in the volume-associated cluster. Two parameters belonged to the length-associated cluster, the VLDI, and the VL–S ratio. The VLDI was correlated negatively with the CVolume and VVolume. The VL–S ratio did not correlate with the CVolume and VVolume.

The CVolume, VVolume, CVI, and SFCT were correlated negatively with the S–V ratio. This result indicated that a large structure, i.e., a large vessel, may increase when the CVolume and VVolume increase. The result was reflected in the VDI increase with the CVolume increase. On the other hand, the VLI also increased with the CVolume increase. The vessel length increase can be associated with the CVolume increase, although the contribution may be smaller than the vessel diameter increase.

The VL–S ratio did not correlate with the VVolume and CVolume. Therefore, the volume of the stroma to the vessel may not vary in healthy eyes.

### The regional variation

The regional variation was measured by 3D analysis previously^20,25^. Cheong et al.^25^ reported that the CVI was higher at the foveal center than at the parafovea, while Zhou et al. reported little regional difference between the regions at the fovea^20^. The CVI in the current study increased at the fovea center. The VL–S ratio also increased at the fovea center, although there was no difference in the VLDI and S–V ratio between the fovea center and the parafovea. These results suggested that the density of the choroidal stroma may decrease at the fovea center. Histopathologically, the connective tissue containing collagen extended outward from the Bruch's membrane like stalactites^26^. At the posterior pole, the intercapillary stroma columns of the choriocapillaris were narrower and shorter than elsewhere. These observations reflected the VL–S ratio decrease at the fovea center. Similarly, the VL–S ratio and CVI were lower in the lower temporal area than in the other quadrants. The stromal volume may be partially reflected in the CVI.

### The relations to aging and AL

Although it is well recognized that the choroidal thickness decreases with age^27,28^ and AL elongation^29^, the changes in the choroidal vasculature are still controversial. Sonoda et al. reported that the luminal/stromal area ratio decreased with age^9^. Ruiz-Medrano reported similar results in the CVI^14^. Zhou et al. reported small changes in the CVI with age in 3D analysis^20^, which was difficult to compare directly to the current study because they used the AL-adjusted CVI for aging analysis. Regarding the choroidal vessel layer, Zhao et al. reported that Sattler's layer thickness decreased with age, while Haller's layer thickness showed little difference during aging^30^. In contrast. Shiihara et al. suggested that the vessel diameter in Haller's layer decreased with age by en-face OCT image analysis^31^.

The current study showed that the CVI decreased with age. The VDI and VLI also decreased, and the VLDI decreased with age. These results suggested that the choroidal vessels might not only shrink in diameter but also shorten in length, implying choroidal vessel remodelling with age. The ultrastructural observations suggested remodelling of the choroidal vasculature^32^. Clinical observation using ICGA revealed choroidal vessel remodelling in pathological conditions, such as radiation^33^, scleral buckling^34^, and photodynamic therapy^35^. The remodelling may be involved in normal aging in the choroid.

Although the AL positively correlated with the VVolume and VDI, the VLDI did not associate with the AL. The choroidal thinning and vessel volume decrease due to the AL elongation may be associated primarily with the reduction in vessel diameter rather than vessel length change. The choroidal layer analysis in non-pathologic myopia suggested that thinning of Sattler's layer was more marked than that of Haller's layer^36^. The result is likely to be inconsistent with our results. We did not analyze the relation of the 3D parameters to the choroidal layer changes. Further studies are needed in the future.

Interestingly, the VL–S ratio did not change with age and AL. The stroma may regress at the same rate as the vessel length reduction. The interaction between the choroidal stroma and the vasculature may be associated with the structural changes of the choroidal vasculature.

### CVI measurement

Zhou et al. reported that the CVI in a 5-mm circle at the fovea was 61.9% based on 3D volume analysis^20^. In the current study, the 3D-CVI was 39.6% in a 4.5-mm circle. There may be several reasons for the difference between the CVI in the previous reports and the current study. First, a minimum of two or more voxels is needed to construct 3D models. Unlike the method of directly binarizing choroidal vessels, the multiscale Hessian method may underestimate the vessel volume because discontinuous voxels are not extracted as a vascular structure. The second reason involves the CVolume segmentation. The current method of CVolume segmentation using the CNN was trained to trace the C/S border, including the suprachoroidal stroma. The outer border of the choroid was the inner vessel wall nearest to the C/S border when it was automatically determined by such a graph-cut method^20,37^. The border did not include the suprachoroidal stroma. As a result, the ratio of the vascular volume to the CVolume decreased in the current study.

## Limitations

This study had several limitations. First, the 3D model in this study was constructed from an OCT silhouette image. Therefore, the other structure of the choroid, such as a “cavern”, maybe the origin of a low-intensity signal^38^. We should be able to detect the blood flow to the vessels correctly. However, it is impossible to detect the blood flow in the large choroidal vessel despite OCT angiography. Second, the crossovers and branching of the vessels may not be reproduced entirely in the 3D model. However, the new parameters derived from the 3D model could not be calculated without modeling. They may be helpful as biomarkers to evaluate the pathophysiology of the choroidal vessels. At a minimum, the results of the aging changes in the vessel diameter in the 3D models were consistent with the previous observations^39^. Third, the pattern of the ROI differed from previous reports. We did not use Early Treatment Diabetic Retinopathy Study (ETDRS) grids but rather grids divided along the horizontal and vertical lines, assuming the histopathologic distribution of a large choroidal vessel running course. Therefore, comparing the current results to previous studies in which ETDRS grids were used was difficult. In addition, the number of subjects was too small to estimate population-based data. Fourth, the line model was generated as an aggregate of points in sectioned vessels passing through an OCT scanning plane in the vessel model. Since the line model was not a true 'line segment,' histopathological validation and reproducibility of the model were not performed in this experimental study. Further studies at this point are needed in the future.

## Conclusions

Three 3D types of the choroidal vasculature models were made from SS-OCT images. The parameters from the models were classified into two groups. The parameters derived from the line model can provide other structural information that differs from the vessel volume. These parameters suggested the structural difference in the choroidal thinning between aging and AL elongation. The parameters from the 3D models warrant further investigation.

## Supplementary Information

Supplementary Table 1.
