# Supplementary material for: Morphometrics in three dimensional choroidal vessel models constructed from swept-source optical coherence tomography images
Source: Sci Rep. 2022 Sep 6;12:15130. doi: 10.1038/s41598-022-17039-9 (PMC9448756; doi:10.1038/s41598-022-17039-9)
Supplement: Supplementary file 1 — Supplementary Table 1. [file 41598_2022_17039_MOESM1_ESM.docx]

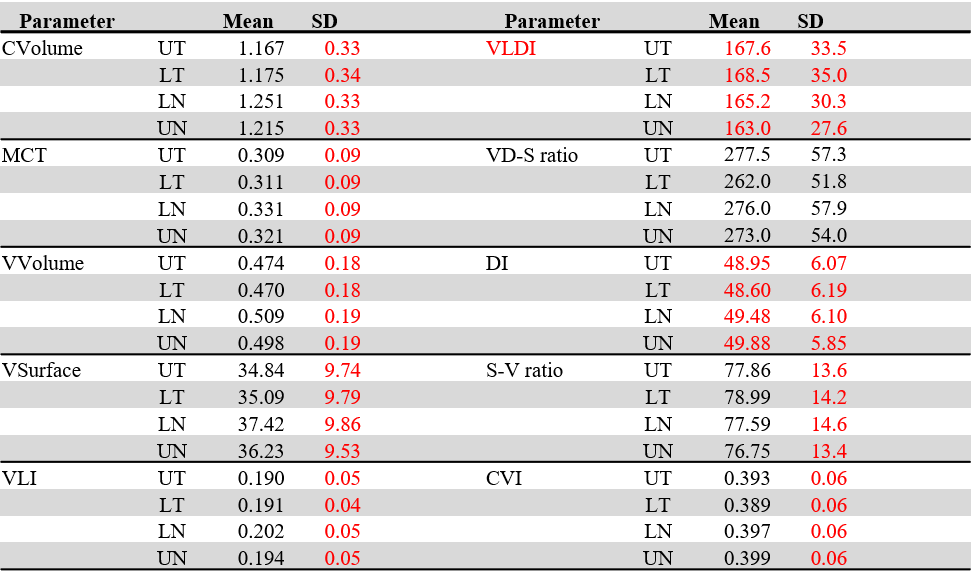


Supplemental Table**. Regional Distribution of Each Measurement by Quadrant.** SD, standard deviation; temp, temporal. The bars indicate a significant difference between the quadrants (P<0.05) calculated by Wilcoxon signed-rank test. UT, upper temporal; LT. lower temporal; LN, lower nasal; UN, upper nasal.
